# Large-Scale Image Analysis for Investigating Spatio-Temporal Changes in Nuclear DNA Damage Caused by Nitrogen Atmospheric Pressure Plasma Jets

**DOI:** 10.3390/ijms21114127

**Published:** 2020-06-10

**Authors:** Xu Han, James Kapaldo, Yueying Liu, M. Sharon Stack, Elahe Alizadeh, Sylwia Ptasinska

**Affiliations:** 1 Radiation Laboratory, University of Notre Dame, Notre Dame, IN 46556, USA; xkapaldo@gmail.com (X.H.); james.kapaldo@gmail.com (J.K.); 2 Department of Physics, University of Notre Dame, Notre Dame, IN 46556, USA; 3 Harper Cancer Research Institute, University of Notre Dame, Notre Dame, IN 46556, USA; yliu12@nd.edu (Y.L.); Sharon.Stack.11@nd.edu (M.S.S.); 4 Department of Chemistry and Biochemistry, University of Notre Dame, Notre Dame, IN 46556, USA; 5 Queen’s CardioPulmonary Unit (QCPU), Department of Medicine, Queen’s University, Kingston, ON K7L 3J9, Canada; elahe.alizadeh@queensu.ca

**Keywords:** atmospheric pressure plasma jets, large-scale imaging, machine learning, cancer treatment, cellular imaging

## Abstract

The effective clinical application of atmospheric pressure plasma jet (APPJ) treatments requires a well-founded methodology that can describe the interactions between the plasma jet and a treated sample and the temporal and spatial changes that result from the treatment. In this study, we developed a large-scale image analysis method to identify the cell-cycle stage and quantify damage to nuclear DNA in single cells. The method was then tested and used to examine spatio-temporal distributions of nuclear DNA damage in two cell lines from the same anatomic location, namely the oral cavity, after treatment with a nitrogen APPJ. One cell line was malignant, and the other, nonmalignant. The results showed that DNA damage in cancer cells was maximized at the plasma jet treatment region, where the APPJ directly contacted the sample, and declined radially outward. As incubation continued, DNA damage in cancer cells decreased slightly over the first 4 h before rapidly decreasing by approximately 60% at 8 h post-treatment. In nonmalignant cells, no damage was observed within 1 h after treatment, but damage was detected 2 h after treatment. Notably, the damage was 5-fold less than that detected in irradiated cancer cells. Moreover, examining damage with respect to the cell cycle showed that S phase cells were more susceptible to DNA damage than either G1 or G2 phase cells. The proposed methodology for large-scale image analysis is not limited to APPJ post-treatment applications and can be utilized to evaluate biological samples affected by any type of radiation, and, more so, the cell-cycle classification can be used on any cell type with any nuclear DNA staining.

## 1. Introduction

In recent years, numerous in vitro studies have shown the considerable anticancer effects of nonthermal atmospheric pressure plasmas in approximately 20 types of malignant cell lines, including lung cancer [1], prostate cancer [2], ovarian cancer [3], osteosarcoma [4], and oral cancer [5]. Furthermore, several in vivo investigations using tumor models of pancreatic cancer [6], glioblastoma [7], melanoma [8,9], ovarian cancer [10], and breast cancer [11] have demonstrated the significant inhibition of cellular growth and tumor damage following atmospheric pressure plasma treatment. The ability of atmospheric pressure plasma jets (APPJs) to inactivate or kill malignant cells relies strongly on the production of a variety of plasma reactive species [12,13]. APPJs synergistically provide free electrons, positive ions, radicals, photons, and electromagnetic fields, which can damage biological targets without elevating the temperature of the treated area [14]. More importantly, plasma treatments in animal models have been reported to selectively damage targeted cancer cells, without affecting surrounding healthy tissues [15,16]. These features suggest that nonthermal atmospheric pressure plasmas may represent a promising alternative to conventional cancer treatments [14,17].

Although some primary clinical studies have previously been performed [18,19,20], the extensive clinical applications of APPJs require more detailed investigations to examine their effects on a variety of cancer cell lines, both in vitro and in vivo [21,22]. There is concern regarding the potential carcinogenic risk and side effects of prolonged clinical use due to the formation of free radicals. These can cause adverse and acute impacts that can present safety risks in long-term APPJ applications [14,23,24]. Also, technical issues, such as the optimal plasma dosage inside tissues, the penetration depth of reactive species, and the distribution of cellular damage, remain poorly understood and require further investigations. A variety of bioanalytical tools and imaging techniques have been used to quantify the induced damage and cellular responses following plasma irradiation, including fluorescence microscopy [25,26,27] and flow cytometry [28]. While these techniques can be utilized to perform routine cellular analyses, each possess both advantages and limitations, in terms of sample preparation requirements, sensitivity, measurable parameters, throughput, and costs. For example, fluorescence microscopy can capture images of small sample regions with high spatial resolution, facilitating the assessment of quantitative morphology [29]. In contrast, flow cytometry can facilitate the analysis of cellular kinetics and cell-cycle phases, but cannot provide spatial information; however, highly sensitive multicolor phenotypic data can be obtained from populations of different cells, within minutes [30].

In the current study, first we explored two dimensional (2D) spatial distributions of damage to deoxyribonucleic acid (DNA) induced by the APPJ treatment of cancer and nonmalignant cells. DNA damage was assessed by measuring double-strand break (DSB) formation in cell nuclei. In the cellular environment, DSBs trigger the phosphorylation of histone H2AX near the break site, resulting in the appearance of γH2AX foci and leading to local changes in the chromatin structure. These modifications are macroscopic structures that can be directly visualized with the assistance of antibody staining inside the cell nuclei.

Second, we developed a large-scale image analysis technique, using machine learning-based cell-cycle classifications, requiring only one staining dye. Generally, the cell cycle is divided into two major phases: interphase, including gap 1 (G1), DNA synthesis (S), and gap 2 (G2), and mitotic (M) phase. During the G1 phase, the cell grows in size at a high biosynthetic rate, producing proteins and copying organelles such as mitochondria and ribosomes to prepare for DNA synthesis (S phase). After DNA duplication, cells enter the second gap phase, G2, during which they grow rapidly and synthesize proteins and organelles in preparation for mitosis. As cells enter the M phase, they stop growing and synthesizing proteins to focus their energy on the complex and highly regulated cell division process. During standard analyses, different dyes are used to stain nuclear DNA in each cell phase. In this study, we used only 4′,6-diamidino-2-phenylindole (DAPI) fluorescence stain to image and classify the cycle of every cell on a coverslip. Although we employed DAPI staining to build a correlation between nuclear DNA versus nucleus size for cell classification, our methodology can be applied to other types of nuclear DNA staining. However, some dyes can also stain mitochondrial DNA outside of the nucleus, and therefore will not be eligible for this method, because this method uses segmentation of nuclei due to their ellipse-like shape.

By combining microscopy images with a machine learning tool, we were able to study the spatial distribution of plasma-induced DNA damage in nuclei within each cell-cycle phase. By mapping the damage distribution over various post-treatment (incubation) times, in both malignant and nonmalignant oral cells, we revealed the spatio-temporal dependence of cellular responses to plasma-influenced regions. These results improve our understanding of APPJ effects on biological targets and their applications in plasma medicine. Furthermore, our proposed methodology for analyzing DNA damage in a large number of irradiated cells could facilitate the quantitative evaluation of the DNA damage caused by any other sources of cellular radiation, with widespread application in radiobiology research.

## 2. Results and Discussion

### 2.1. Computational Analysis

**Figure 1:**
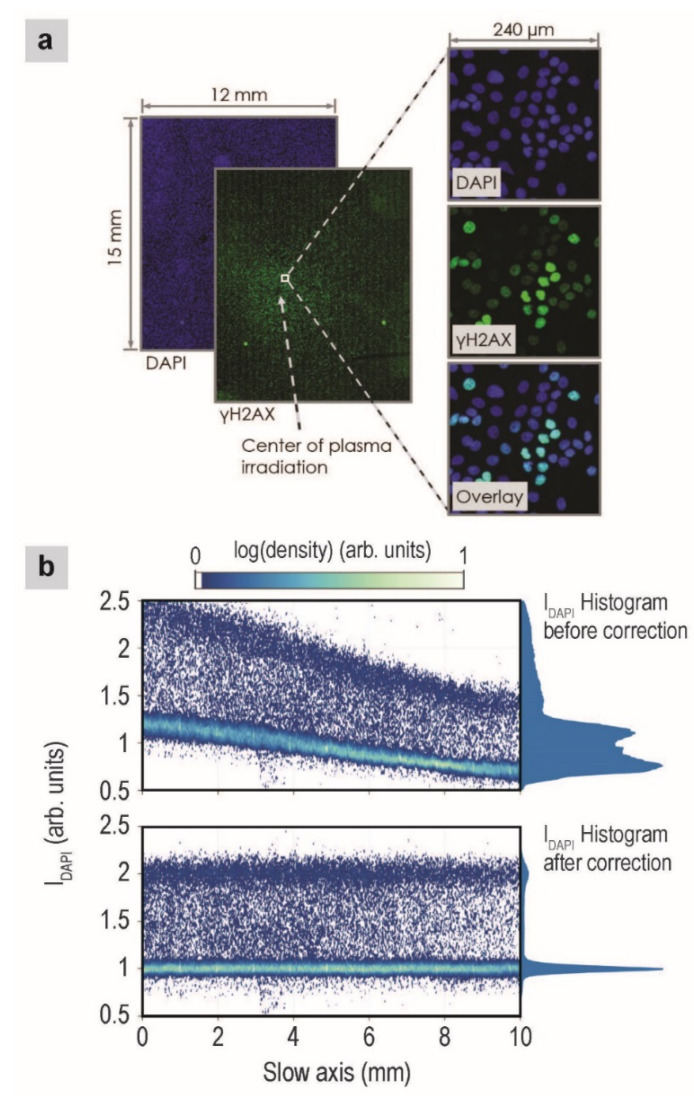
(**a**) Representative fluorescence images of cells, after 2-min plasma treatment and 1-h incubation. Green and blue fluorescence signals correspond to γH2AX and DAPI, respectively. (**b**) Effects of background correction. Plots show the nuclear DAPI intensity along the slow axis of the fluorescence slide scanner. Colors encode the nuclear density (along the slide scanner’s fast axis). A histogram of the nuclear DAPI intensity is indicated to the right of each plot. The top and bottom plots show data before background correction and after flattening and stripe artifact removal, respectively.

Figure 1a shows representative fluorescence images of nuclear DNA (denoted as the DAPI channel) and DSBs in nuclear DNA (denoted as the γH2AX channel) on a large scale. The slide scanner’s movements followed a zig-zag pattern, first scanning the entire length of the coverslip along one direction (called the fast axis), then taking a short step to the side (called the slow axis), before again scanning the entire length of the coverslip in the opposite direction, until the whole surface was scanned.

The image analysis is comprised of four primary steps, including nuclei segmentation and background/foreground correction, feature extraction, machine learning to classify the cell phase, and quantification of DNA damage.

#### 2.1.1. Nuclei Segmentation and Image Correction

Nuclei segmentation was achieved by filtering the DAPI images with a Gaussian blur filter (σ = 1) and then using an adaptive, log-weighted Otsu threshold [31]. This method successfully segmented all nuclei that were not in contact with other nuclei; however, in our studies, the initial confluence of the cells was 90%, and confluence increased during the incubation time, resulting in many overlapping nuclei. These overlapping nuclei needed to be further segmented for additional computational analyses, which was accomplished by applying a short-range attraction and a long-range repulsion (SALR) clustering algorithm [32], in which we identified the center of each nucleus following a geometric partitioning algorithm to segment the overlapping nuclei [33].

In addition, intensity correction of the image background and foreground is crucial for successful analysis. The image foreground represents all regions (pixels) containing nuclei; these regions were determined by the segmentation in the previous step. Likewise, the image background represents all regions not containing nuclei. Three primary causes of uneven background/foreground intensities were identified across our images: uneven fluorescence staining, which results in slowly changing background/foreground intensities; differing amounts of microscope illumination/collection, which can be caused by improper focusing across the whole coverslip; microscope calibration errors, which led to the appearance of bright stripes along the fast axis of the scanned images.

Using the background region, we computed the spatially variable background intensity for each channel and removed it from the image. After this background flattening, we computed the background stripe artifacts and removed them. A representative stripe artifact can be observed in the large γH2AX image in Figure 1a. DAPI channel foreground correction was performed before the foreground correction of other channels. Two bands of DAPI intensities are observed in Figure 1b, which likely correspond to the G1 and G2 phases, since cells in the G2 phase contain twice as much DNA as cells in the G1 phase and because cells spend the most time in the G1 and G2 phases. We fitted these two bands with locally weighted linear fits (lowess) to flatten the G1 and G2 bands and position them at DAPI intensity values of 1 and 2, respectively. Figure 1b shows the nuclear DAPI intensities across the slow axis of the scanner, before (top) and after (bottom) correction. After the correction, the G1 and G2 DAPI bands are flat and positioned correctly. The foreground correction for the γH2AX channel was performed with caution, as the intensity values should not be flat because plasma treatment causes localized DSBs in the nucleus. We first qualitatively selected all G1 cells, using the corrected DAPI intensities from above (cells with DAPI values in the range of 0.7–1.3). Using these cells, we fitted a surface to the locally varying data in the bottom 2%–4% of the γH2AX intensities and then subtracted this surface from the data. This process will correctly flatten the γH2AX channel as long as some of the cells in a large region of the image (~1 mm^2^) are not influenced by the plasma, which is true in our images based on the fact that the maximum damage ratio of G1 cells is 50% (as described below) and therefore, the bottom 2%–4% of γH2AX intensities represent undamaged cells.

#### 2.1.2. Feature Extraction

From each nucleus, we extracted a set of features from the DAPI channel for classifying cell-cycle phases. The features describe the nucleus shape, intensity, radial (shell) intensity, Haralick texture [34], and granularity. The Haralick and granularity features were computed using the same method as the commonly used CellProfiler software (version 2.1.1) [35]. The nuclear shapes were described, in part, using Fourier descriptors [36]. A list of the extracted features can be found in Appendix A. From the γH2AX channel, we extracted intensity features only. The integrated intensity of the γH2AX channel for each nucleus is proportional to the quantity of DSBs in each nucleus.

The code for feature extraction was written to compute the features of multiple nuclei in parallel on a graphical processing unit (GPU), in contrast with CellProfiler, which processes images in parallel on central processing units (CPUs) and processes nuclei features in serial. Extracting the nuclei features on a GPU results in a significant decrease in processing time when a large number of CPUs are not available. When applying our code during some basic tests, the entire processing time for the images (including segmentation and correction, which also used GPU acceleration), including feature extraction, required approximately 40–60 min per image, for both the DAPI and γH2AX channels, based on approximately 3 GB per channel. When using four CPUs, the same processing in CellProfiler took 2–3 h, without the application of SALR clustering for the accurate partitioning of nuclei clumps [35]. In addition, when using a simple watershed-based partitioning of overlapping cells, which is more similar to the method used by CellProfiler, our processing time is shortened to 20–30 min per image. The code was written in MATLAB (The MathWorks, Inc., Natick, MA, USA), and the hardware used for comparisons were an Nvidia GTX770, with 2 GB of memory, and an Intel 4 core i7-4790, 3.6 GHz.

#### 2.1.3. Cell-Cycle Classification

**Figure 2:**
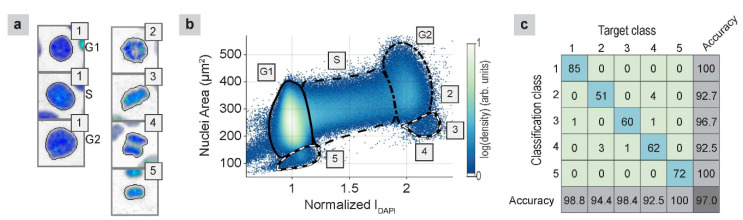
(**a**) Examples of nuclei from each classification group; 1: interphase, 2: prometaphase, 3: metaphase, 4: early anaphase, 5: late anaphase, telophase, and early G1. The images are 34 µm × 34 µm, and they are modified to show a white background, for ease of viewing. (**b**) Nuclear area versus DAPI intensity (color gives nuclear density), with classification contours that enclose regions that contain primarily G1 (solid), S (dashed), G2 (dot-dashed), and M (black/white dash) phase cells. The right M contour primarily contains classes 3 and 4, whereas the left M contour primarily contains class 5. (**c**) Confusion matrix of the test set, based on the classification of different cell classes.

The cell-cycle phase was classified using two steps. During the first step, supervised machine learning was used to classify five visually distinct classes. Class 1 included interphase (G1, S, and G2), and the other four classes were derived from the different mitosis stages, including prometaphase (class 2), metaphase (class 3), early anaphase (class 4), and late anaphase, telophase, and early G1 (class 5). Examples of these classes are presented in Figure 2a. We manually classified approximately 500 nuclei in each class to create a balanced dataset, which was divided into training, validation, and test sets at a 0.7/0.15/0.15 ratio. The employed learning method was a shallow neural network, with two hidden layers (sizes 30 and 10), and a softmax layer to output the final classifications. The training used early stopping with the validation data to prevent overtraining. The confusion matrix on the test dataset is shown in Figure 2c, indicating that the overall classification accuracy was 97%. The network creation and training were all performed using MATLAB built-in routines, namely the “patternnet” and “train” functions.

During the second step, the interphase cells were classified using a mixture-of-Gaussians model, with a uniform background [37], using the nuclear area and DAPI intensity (Figure 2b). The classification used seven Gaussians, equally spaced along the line connecting the center of the G1 and G2 peaks (Figure 2b), and an eighth Gaussian, located on the G1 peak. This classification of interphases was confirmed by experiments using an anti- chromatin licensing and DNA replication factor 1 (CDT1) antibody to label G1 phase cells and 5-ethynyl-2’-deoxyuridine (EdU) to label S phase cells (results not shown). Figure 2b shows the distribution of the nuclear area versus the DAPI intensity, along with contours that denote regions containing cells in the G1, S, G2, and M phases. From this plot, we visualized the cell progression through the entire cell cycle. After the G1 phase (I_DAPI_ = 1), cells enter the S phase, during which they duplicate their DNA (I_DAPI_ = 1 → 2). Then, they enter the G2 phase and prepare for mitosis (I_DAPI_ = 2). As they enter mitosis, they begin to condense, and their areas become smaller (I_DAPI_ approximately 2.3, area approximately 250 µm^2^). During anaphase, the image segmentation splits the two halves into distinct objects, resulting in an area and a DAPI intensity equal to half of the previous values (I_DAPI_ approximately 1.1, area approximately 120 µm^2^). The nuclei then start to grow and reenter the G1 phase.

#### 2.1.4. Damage Quantification

During the final step, the damage to each nucleus is defined as a fraction of the total damaged DNA, which can be computed as the ratio between the γH2AX and DAPI intensities, I_γH2AX_/I_DAPI_. To quantify the susceptibility of each cell phase to damage induced by the plasma jet, we used the damage ratio (DR), which indicates the number of cells with damage above a specified threshold divided by the total number of cells.

### 2.2. APPJ Irradiation

The above-described computational procedure for large-scale image analysis, with machine learning, was used to acquire the spatio-temporal distributions of DNA damage in malignant and nonmalignant cells after treatment with a nitrogen APPJ. The obtained images for two cell lines are presented, and the biological implications of plasma treatment are briefly discussed in the following subsections. These results are presented to illustrate the usefulness of the procedure for assessing the biological effects after plasma treatments, and certain conclusions have been derived; however, exploring the biological mechanisms induced by APPJs is not the focus of this study.

#### 2.2.1. APPJ-Irradiated Malignant Cells

**Figure 3:**
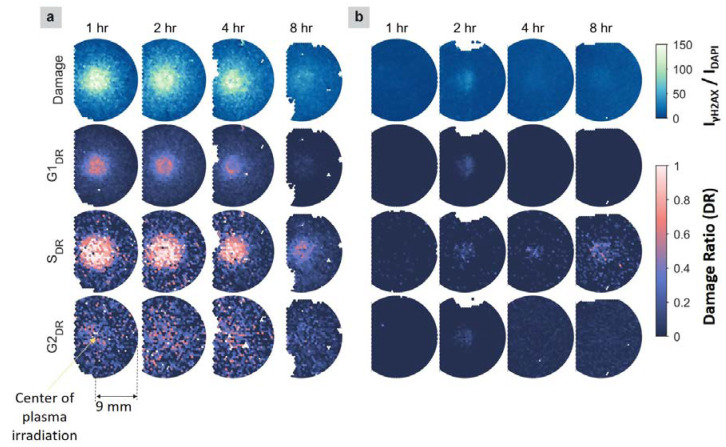
2D distributions of the damaged DNA fraction in each nucleus (top row) and the damage ratios (DRs) for cells in G1, S, and G2 phases (rows 2–4), based on γH2AX staining in malignant cells (**a**) and nonmalignant cells (**b**) grown on coverslips. The damage threshold used to determine the DR was 75 (noting the damage range is 0–150). Each circular distribution, with a diameter of 18 mm, is centered on the treatment locations. Locations with white patches indicate regions without cells or blurry regions of the image, which were discarded. The circles for the cancer cells are cut off on the left side, due to a limitation of the slide scanner.

The first row of Figure 3a shows the 2D distributions of cancer cell damage after different incubation times. These distributions were obtained after subtracting the median damage value of the flow control cells, which did not show any effects following nitrogen flow treatment alone. After 1-h incubation, cells were observed to be damaged near the plasma jet treatment region, and the damage decreased radially outward. Damage that extends beyond the plasma jet treatment region may be caused by the following: (1) the diffusion of the plasma species above the liquid surface; (2) the diffusion of the reactive species in the liquid, induced by the plasma treatment; (3) cell−cell communications, which may contribute to the damage of bystander cells near the treated cells [38]. Similarly, an enlarged affected area has been observed in previous studies [5,15,39].

**Figure 4:**
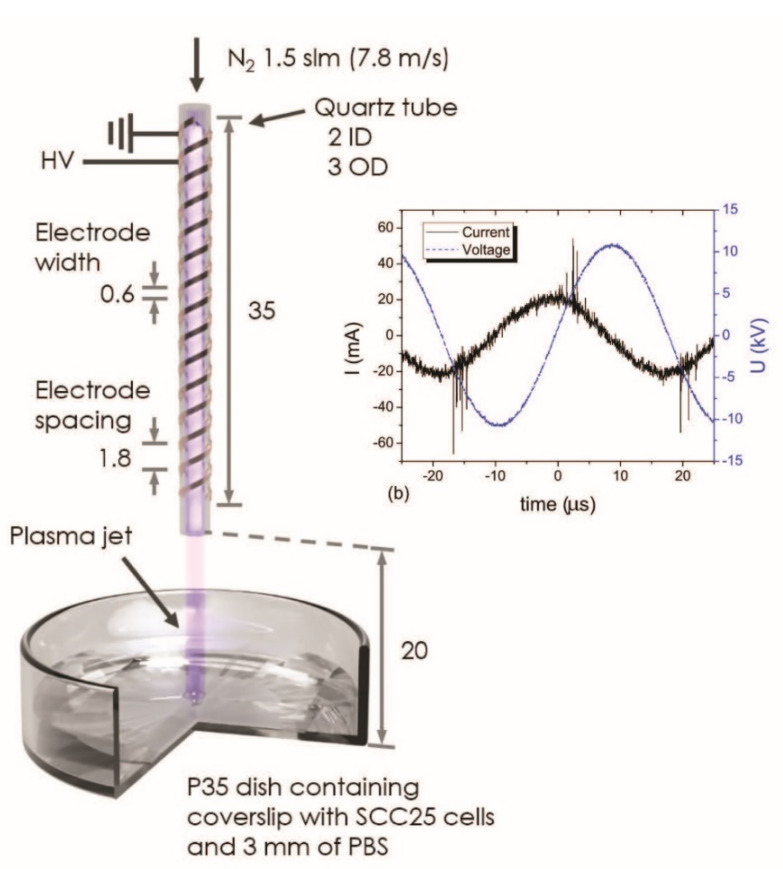
A schematic diagram of the nitrogen APPJ source used to treat cultured cells on coverslips. All dimensions are in mm. The inset shows the high voltage (HV) and current of alternating current (AC) waveforms of the nitrogen APPJ discharge.

More importantly, the maximum damage was observed at locations approximately 1 mm away from the treatment center, as shown in cancer cells after 2 h of incubation, resulting in a ring-shape pattern centered on the treatment location. The radius of this ring was similar to the inner radius of the quartz tube orifice, which was 1 mm (Figure 4), suggesting that the most prominent damaging effects were caused by species located at the interface of the plasma jet and the surrounding air. At this interface, highly reactive species have formed that interact with cells, causing DSBs. For example, one highly reactive species produced at the interface is nitric oxide (NO) [5,40], which can cause DNA strand breaks via the production of other reactive nitrogen species (RNS), such as ONOOˉ, HNO_2_, and N_2_O_3_ [41]. NO can also trigger the production of intracellular reactive oxygen species (ROS), which can initiate various pathways, including apoptosis. Similar observations of ring-shaped regions have also been reported in previous studies, such as the inactivation patterns of bacteria, the distributions of reactive species, theoretical modeling [42], and for a gas-shield, helium-based APPJ, during interactions with cancer cells [43]. After 8 h of incubation, the measured damage decreased, which was likely due to cell detachment. Those cells that suffered from severe damage could not recover through repair processes and, consequently, undergo cell death. As a result, the dead cells detach from the coverslip and are removed during the washing steps of immunofluorescence procedures. Similar cell detachment after plasma treatment has been observed in other studies [44,45].

Furthermore, cancer cells in different cell-cycle phases have been found to respond with different sensitivities to plasma treatments. As shown in Figure 3a, after 1 h of incubation, almost 100% of S phase cells were damaged at the treatment region, whereas a much smaller proportion of G1 and G2 phase cells showed damage. In addition, the damaged area for S phase cells was found to be much larger than those for cells in the G1 and G2 phases. These results imply that S phase cells are more sensitive (susceptible) to damage induced by plasma treatment than cells in the G1 and G2 phases. During the S phase, DNA replication requires the exposure of a single-stranded portion of DNA near the replication fork, which results in an increased vulnerability to external attacks compared with other phases. Such attacks can be due to reactive species that are solvated/generated in the liquid above the cells during the treatment [46], which generally extend radially outwards beyond the location where the plasma jet makes direct contact with the liquid, through diffusion in the liquid phase or the transport of the gas phase plasma species across the liquid surface by the nitrogen gas flow. The damage caused during DNA replication may also be associated with the production of intracellular ROS/RNS, as a cellular response to plasma treatment [47].

Additionally, the communication between treated cells and adjacent cells (i.e., bystander cells) can initiate cellular damage pathways in the bystander cells, similar to those initiated in treated cells [38]. Therefore, DNA damage in a cell layer can propagate radially outwards from the treatment region. Notably, G1 phase cells also showed circular damage patterns, with a damage ratio of approximately 50% after 1 h of incubation, whereas the damage ratio observed for G2 phase cells was much lower and not prominently localized at the plasma treatment region. These results are different from the observations reported previously [28], in which no significant γH2AX signals were found for either the G1 or G2 phases. These differences may be due to the different plasma sources used (i.e., helium plasma jet in the previous study [28], compared with the nitrogen plasma jet in our study), as the composition and distribution of plasma species may differ, resulting in different levels of damaging effects.

#### 2.2.2. APPJ-Irradiated Nonmalignant Cells

In contrast with the malignant cell line, plasma-treated nonmalignant cells did not show damage after 1 h of incubation (Figure 3b). After 2 h of incubation, the distribution of total damage in nonmalignant cells showed a circular pattern, with a much lower intensity than that observed in cancer cells (<1/5 the maximum value). This result suggested that under our experimental conditions, nonmalignant cells were minimally influenced by the plasma treatment. There are several factors: biological (e.g., gene expression, member structure, tolerance to oxidative stress) and experimental (e.g., dose and type of delivered radicals produced in APPJ from different plasma sources) factors reported that could contribute to different responses in various cell lines [14]. Because experimental conditions for both cell lines in this study are the same, the massive difference in damage susceptibility between two cell lines following plasma treatment is due to their biological differences. The observation that plasma-treated nonmalignant cells displayed mild damage at a later time (2 h of incubation compared with 1 h of incubation for cancer cells) suggested that the two types of cells respond at different rates. Therefore, to accurately compare the plasma-induced effects on different types of cells, the cellular responses must be monitored over time, instead of using a single time point after treatment. However, more studies are needed to assess the APPJ effectiveness in biological targets and to determine the mechanisms through which plasma interacts with cells, which are outside of the scope of this study.

## 3. Materials and Methods

A schematic diagram of the APPJ source and the experimental setup is illustrated in Figure 4. A detailed description of the plasma source used in this study has been previously reported [5]. The APPJ was ignited with a 22.4-kV peak-to-peak voltage and a 59-mA peak-to-peak leaking current, at 28 kHz (Figure 4). Ultrahigh, pure, 5.0-grade nitrogen (purity of 99.999%, Airgas, Radnor, PA, USA) was used as the feed gas, with a flow rate of 1.5 standard liters per minute (slm), corresponding to a gas speed of approximately 7.8 m/s. The gas flowed through a quartz tube, with an inner orifice of 2 mm. Oral cancer cells originally derived from squamous cell carcinoma of the tongue (SCC25) was obtained from American Type Culture Collection (ATCC, Rockville, MD, USA) and grown to approximately 90% confluence (approximately 10^5^ cells·cm^−2^), on coverslips, in p35 dishes. Prior to plasma exposure, the cell culture medium in the p35 dish was replaced with 2.4 mL phosphate-buffered saline (PBS, 1 X, Mediatech. Inc., Manassas, VA, USA), forming a 3-mm-thick liquid layer above the cells. The detailed recipes for the cell culture medium used with cells in this study are listed in Appendix B. During the treatment, the dish was placed 2 cm below the quartz tube orifice (Figure 4). A plasma treatment time of 2 min was selected based on our previous study as the optimal treatment time for the detection of DNA damage without causing excessive buffer evaporation [5]. After 2 min of treatment, the PBS was replaced with 2 mL fresh culture medium, and the dishes were transferred to an incubator for 1, 2, 4, and 8 h. The samples were incubated at 37 °C, in an atmosphere of > 95% humidity and 5% CO_2_. Before image analysis, cells were fixed, permeabilized, and blocked, including PBS washes between each step, as described previously [5]. For imaging purposes, anti-phospho-histone H2AX (Ser139) antibody (Mouse, EMD Millipore Corp.) and goat anti-mouse IgG (H+L) (Alexa Fluor 488 from Thermo Fisher Scientific Inc., Waltham, MA, USA) were applied, to evaluate the DSBs in nuclear DNA. Fluorescent DAPI Mounting Solution (Vector Laboratories Inc., Burlingame, CA, USA) was used to stain nuclear DNA, and then the DNA contents were visualized and quantified.

We developed this large-scale image analysis method to facilitate cell-cycle classifications using only DAPI fluorescence. Cell-cycle classifications were confirmed by experiments using anti- CDT1 (Abcam plc.) antibody, which label G1 phase cells, and EdU (Thermo Fisher Scientific Inc.), which label S phase cells.

To compare the effects of plasma treatments between two cell lines, the same preparation and treatment procedures were performed for oral nonmalignant cells. Telomerase reverse transcriptase-immortalized normal oral keratiocytes (OKF6/T) cell were the generous gift of James Rhinewald, Brigham and Women’s Hospital, Harvard Institutes of Medicine (Boston, MA, USA). Additionally, in the case of SCC25 and OKF6/T cells, two groups of samples were prepared using the same incubation times, including one group treated with nitrogen flow but no plasma irradiation and one group that received no treatment, which were used as control groups.

When the cells were not actively in culture, the cells were frozen and preserved for long-term storage. The procedures for cell culture and for freezing and thawing cells conducted in this work are provided in Appendix C and Appendix D, respectively.

To obtain cellular data, the coverslips were scanned with an Aperio fluorescence slide scanner (Leica Microsystems, Wetzlar, Germany), using the DAPI and Alexa Fluor 488 (denoted as γH2AX) channels, at 20× magnification. The immunofluorescence staining procedures are described in Appendix E.

## 4. Conclusions

We introduced a large-scale image analysis method, combined with machine learning, to study the spatial distributions of plasma jet-induced nuclear DNA damage over time in two cell lines from the oral cavity. The analysis included nuclear segmentation utilizing SALR clustering, the background and foreground correction of images, nuclei feature extraction, and cell-cycle classification. We demonstrated that the application of fluorescence microscopy using only DAPI staining (or any other dye for nuclear DNA staining) could achieve successful cell-cycle classification. This classification system not only preserves spatial information but also allows the discrimination of M phase cells from interphase cells, which flow cytometry often fails to accomplish.

By using this analysis method, we were able to visualize that the 2D DNA damage distributions depend on the different cell-cycle stages at various incubation times. Therefore, to realize the efficacy of plasma treatments, post-treatment time-dependent assessments are necessary to monitor the cellular responses in various irradiated cancerous cell lines and their nonmalignant counterparts. Determining the spatial and temporal distributions of nuclear DNA damage in plasma-irradiated cells can have significant impacts on revealing the molecular mechanisms of plasma effects. The robustness and versatility of this method will be beneficial for a more systematic and rigorous assessment of the strengths, such as selective targeting features and weaknesses, such as side effects of plasma clinical applications. Furthermore, our proposed methodology can be used to quantitatively examine the effects of other types of radiation on biological samples.

## Figures and Tables

**Figure A1:**
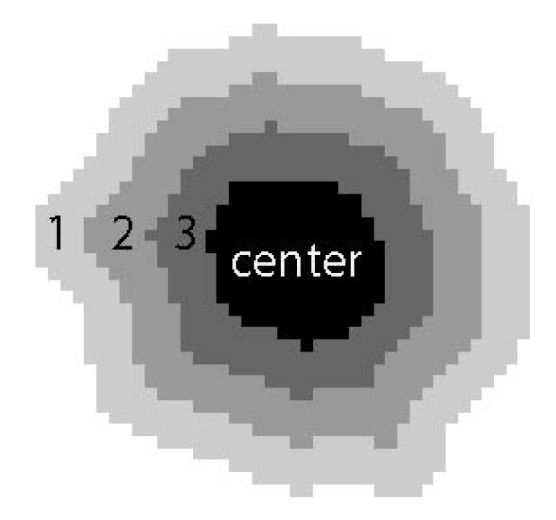
Radial intensity features. Example of the 3 rings with a width of 4 pixels and the center region. In each of these four regions, the mean and standard deviation of the intensity are computed.

## Appendix A. Extracted Features

A list of the extracted features is given in Table A1, following a few notes. The number of Fourier descriptors for description nuclei shape was 20. Haralick textures [34] were computed using periods of 3 and 7 pixels and the features were averaged over the four computation directions (0°, 45°, 90°, and 135°). The granularity spectrum, length of 7 pixels, was computed after removing the background (morphological opening using a disk with radius of 10 pixels) using a background structuring element with radius of 10 pixels. The radial intensity features used the distance transform of a nucleus segmentation mask to extract intensity features from rings around the nucleus with different widths and radii. We computed the radial intensity values for three rings with a width of 4 pixels, as well as the center region of the nuclei as shown in Figure A1. The only features extracted from the γH2AX channels were Intensity features.

**Table A1:** List of Extracted Features.

Feature Group	Feature Name
Shape	Area Major axis length Minor axis length Eccentricity solidity form factor Fourier descriptors (20)
Intensity	Integrated mean deviation Kurtosis
Radial intensity (N3 W4)	Mean Standard deviation
Haralick texture (3, 7)	Contrast Correlation Difference entropy Difference variance Energy Entropy Information measures correlation 1 Information measures correlation 2 Inverse difference moment Sum average Sum entropy Sum of squares variance Sum variance
Granularity	----

## Appendix B. Cell Culture Medium Recipes

The preparation of cell culture medium is required to be carried out in a laminar flow hood in a sterile condition. All the components of the medium are required to be filtered. The filter used in this study is with a polyethersulfone microporous membrane which has a pore size of 0.2 μm.

### Appendix B.1. SCC25 Cell Culture Medium: ~500 mL

- Dulbecco’s modified Eagle medium (DMEM): 250 mL;
- Ham’s nutrient mixture F-12 (F-12): 250 mL;
- Fetal bovine serum (FBS): 50 mL;
- Penicillin streptomycin (Pen Strep): 5 mL;
- Amphotericin B: 0.5 mL.

### Appendix B.2. OKF6/T Cell Culture Medium: ~500 mL

- Keratinocyte-serum free medium (SFM): 500 mL;
- Epidermal growth factor 1-52 (EGF 1-53, 0.03 μg/μL): 3.3 μL;
- Bovine pituitary extract (BPE, 10 mg/mL): 1.25 mL;
- Calcium chloride (CaCl_2_, 2M): 77.5 μL;
- Penicillin streptomycin (Pen Strep): 5 mL.

## Appendix C. Cell Culture Procedure

The cell culture procedures are required to be carried out in a laminar flow hood in a sterile condition.

### Appendix C.1. Primary Culture

This process is usually carried out every other day before the cells reach confluence. Fresh medium is needed to be prewarmed (37 °C) before use.

- Remove the cell culture medium in the p100 dish and wash the cells with PBS (1X) twice.
- Add 12 mL fresh medium in the dish.
- Return the dish to the incubator.

### Appendix C.2. Subculture

This process is required when the cells are over 90% confluence. Fresh medium is needed to be prewarmed (37 °C) before use.

- Remove cell culture medium in the p100 dish and wash the cells with PBS (1X) twice.
- Add trypsin 2 mL (2 mL is enough for covering the surface of a p100 dish) and transfer the cell dish into an incubator for 3 min.
- Observe the cells under a microscope to check if the cells are detached from the dish surface. If not, prolong the incubation time or tab the side of the dish gently to expedite cell detachment.
- Add 4 mL fresh medium to the dish (make sure this amount is at least twice the volume of the added trypsin). Disperse the medium by pipetting the cell suspension over the cell layer surface several times.
- Harvest all the cell suspension into a 15 mL centrifuge tube.
- Centrifuge the tube at 1200 rpm for 2 min.
- Remove the supernatant above the cell pellet. Add 4 mL fresh medium, resuspend the cell pellet by pipetting and mixing.
- Add 11.5 mL fresh medium in a new p100 dish.
- Distribute 0.5 mL cells suspension into the new dish. As a result, the cells are seeded with 1:8 ratio in 12 mL medium in the p100 dish. The seeding ratio can be varied according to experimental needs. With 1:8 ratio dilution, SCC25 cells usually become confluent in approximately 4 days, and OKF6/T in approximately 6 days.
- Move the dish in four directions (forward, backward, left, and right) horizontally several times before transferring it to the incubator. This procedure will facilitate the cells to be seeded uniformly throughout the whole dish area.

## Appendix D. Freezing and Thawing Cell Procedures

### Appendix D.1. Freezing

This process can be carried out when the cells are over 90% confluence in p100 dishes. Freezing medium is needed to be prepared at 2–8 °C before use. Due to freezing medium contains dimethyl sulfoxide (DMSO), which is known to facilitate the entry of organic compound into human tissues, it is necessary to handle freezing medium carefully with proper personal protective equipment (PPE). A freezing container with isopropanol is needed to be prepared in room temperature. Once the isopropanol chamber is used five times for freezing, the isopropanol in the container must be replaced with fresh isopropanol.

- Conduct the first 6 steps in Appendix C.2.
- Remove the supernatant above the cell pellet. Add 1.2 mL prewarmed freezing medium, resuspend the cell pellet by pipetting and mixing.
- Dispense the cell suspension in two cryogenic storage vials with 0.5 mL each.
- Place the vials in the isopropanol chamber and store it at −80 °C overnight.
- Transfer the vials to a storage unit in a liquid nitrogen tank.

### Appendix D.2. Thawing

The newly added medium should be changed the next day after the cells being thawed following the steps below. This is to remove the hazard of cryoprotective agents such as DMSO that present in the freezing medium. As mentioned in Appendix D.1, the freezing medium should be handled with care.

- Prepare a p100 dish with 10 mL fresh prewarmed cell culture medium in a sterilized laminar flow hood.
- Thaw the frozen cells rapidly (<1 min) by gently swirling the vial in a 37 °C water bath until the ice remains a little bit in the vial. At this step, a face mask or goggles are required in addition to gloves for PPE for the hazard of vial explosion.
- Sterilize the vial surface with 70% ethanol before transferring to the hood.
- Transfer the cells into the prepared dish filled with fresh medium with micropipette. Add 10 mL more fresh medium to the dish and gently mix with the cells.
- Move the dish in four directions horizontally several times before transferring it to the incubator.

## Appendix E. Immunofluorescence Staining Procedures

### Appendix E.1. Single Antibody (AB) Staining

Single antibody staining uses one primary antibody and one secondary antibody to stain the cells. The cells are grown on square coverslips in p35 culture dishes. Paraformaldehyde (PFA, from Electron Microscopy Sciences, PA, USA), Triton X-100 (from American Bioanalytical, MA, USA), and bovine serum albumin (BSA, from Gold Biotechnology, MO, USA) are previously prepared with desired concentrations in 1X PBS.

Day 1:

(1) Wash cells with PBS (1X) twice.
(2) Fix the cells by adding 1 mL PFA (4%) to the dish for 20 min.
(3) Remove PFA to a biowaste container. Since PFA is toxic, it should not be discarded with other conventional biowaste solutions.
(4) Rinse the cells with PBS (1X) twice.
(5) Permeabilize the cells with Triton X-100 (0.3%) for 5 min.
(6) Remove the Triton X-100 and wash the cells with PBS (1X) three times.
(7) Apply blocking solution BSA (3%) to the cells for 1 h.
(8) Prepare primary AB solution. For example, anti-phospho-histone H2AX needs to be diluted with a ratio of 1:250 in 3% BSA. The amount of antibody needed can be calculated as follows:

(A1) $$V_{AB}\left(\mu L\right)=\frac{V_{total}}{dilution\;factor}=\frac{75\mu L\times N_{sample}+30\mu L}{250}$$

(A2) $$V_{BSA}=V_{total}-V_{AB}$$

where V_AB_ is the volume of antibody from the stock, V_total_ is the volume of the final solution, V_BSA_ is the volume of the 3% BSA as a solvent. The volume of the final solution applied to each coverslip for antibody staining is 75 μL. There is an extra volume of 30 μL in case of bubble formation inside the solution during pipetting and mixing.

(9) Prepare a flat parafilm sheet as a platform for AB incubation. The film can be placed at the bottom inside a square plastic container (e.g., 10 × 10 × 15 mm square petri dish with lid, SKS Science Products). The inside of the lid is covered with a wet Kimwipe tissue, which will facilitate maintaining the moisture during AB incubation process.
(10) Carefully add 75 μL primary AB each at a set of dispersed locations (the number of locations equals to N_sample_) on the parafilm avoiding bubbles while pipetting.
(11) Take out the coverslips immersed in BSA from the dishes.
(12) Flip the coverslips enabling the face of the coverslip that having cells facing down towards the parafilm. Rest one side edge of the coverslip on the film, then carefully lower down the other side of the coverslip, till the cells on the entire coverslip are in contact with the solution. Make sure there are no bubbles in between the coverslip and the parafilm.
(13) After finishing the previous step for all the samples, put on the wet-tissue-covered lid. Transfer the container to a 4 °C room overnight.

Day 2:

(14) Fill coverglass staining jars with 1X PBS. Each jar can contain four coverslips vertically.
(15) Transfer the coverslips from the container to the staining jars. Make sure to record the orientation of the coverslip faces that have cells.
(16) Remove the PBS inside the jars. Fill the jars with fresh PBS and put the jars on a shaker (Orbit shaker, Barnstead Lab-Line, 600 rpm) for 5 min. Repeat this step one more time.
(17) Prepare secondary AB solution. For example, Alexa Fluor 488 goat anti-mouse needs to be diluted with a ratio of 1:400 in 3% BSA. The amount of the antibody needed can be calculated in the same method as Equation (E1) with a dilution factor of 400. During preparation, light exposure to the secondary AB must to be kept as short as possible.
(18) Repeat step (9), (10) with secondary AB, (11) with taking out coverslips immersed in PBS from the jars, and (12).
(19) After finishing the previous step for all samples, close the container with the wet-tissue-covered lid. Keep the container in dark for 20 min.
(20) Repeat step (15). Similar as the step (16), wash the coverslips in PBS on a shaker four times with 5 min each. Repeat washing the slides once with reverse osmosis (RO) water on a shaker for 5 min. Keep the entire washing process in dark.
(21) Take out the coverslips from the jars. Place the coverslips on a Kimwipe tissue with the cells facing up. Air dry the coverslips in dark.
(22) Prepare glass microscope slides with 20 μL DAPI droplets on top. One DAPI drop per slide is preferred. Be careful to protect DAPI from light.
(23) Repeat step (12) for staining the cells with DAPI solution. Press the coverslips on the slides vertically to remove extra DAPI with Kimwipe tissues.
(24) If the DAPI solution used is a soft-mounting medium, apply clear nail polish on four edges of the coverslips to seal the slides. If the DAPI used is a hard-mounting medium, no further sealing process is needed.
(25) Store the slides in a slide storage box in a 4 °C room in dark.

### Appendix E.2. Double Antibody Staining

Double staining (DS) uses two primary antibodies and two secondary antibodies with different fluorescence emission ranges to stain two different parts of a cell. The procedures of double antibody staining are the same as the single antibody staining except following modifications of the step (8) and the step (17) to be (26) and (27), respectively:

(26) Prepare AB solution with two primary ABs. For example, anti-phospho-histone H2AX and cleaved caspase-3 antibody need to be diluted in 3% BSA with a ratio of 1:250 and 1:400, respectively. The amount of antibody can be calculated as follows:

(A3) $$V_{AB1}\left(\mu L\right)=\frac{V_{total}}{dilution\;factor\;1}=\frac{75\mu L\times N_{sample}+30\mu L}{250}$$

(A4) $$V_{AB2}\left(\mu L\right)=\frac{V_{total}}{dilution\;factor\;2}=\frac{75\mu L\times N_{sample}+30\mu L}{400}$$

(A5) $$V_{BSA}=V_{total}-V_{AB1}-V_{AB2}$$

where V_AB1_ and V_AB2_ are the volumes of the two antibody solutions needed.

(27) Prepare AB solution with two secondary ABs. For example, both Alexa Fluor 488 goat anti-mouse and Alexa Fluor 594 goat anti-rabbit need to be diluted in 3% BSA with the ratio of 1:400. The amount of each antibody can be calculated in the same method as Equations (4) and (5) with a dilution factor of 400.

### Appendix E.3. S Phase Cell Staining with Click-iT EdU Alexa Fluor 647 Imaging Kit and Single AB

Cells are grown on square coverslips in p35 culture dishes. PFA, Triton X-100, and BSA are previously prepared with desired concentrations in 1X PBS. Different stock solutions are previously prepared according to the manufacture protocol [48].

(28) Incubate cells with fresh medium containing 10 μM EdU for 2.5 h.
(29) Wash cells with PBS (1X) twice.
(30) Fix the cells by adding 1 mL PFA (4%) for 15 min.
(31) Remove PFA to a biowaste container. Since PFA is toxic, it should not be discarded with other conventional biowaste solutions.
(32) Wash cells with 1 mL 3% BSA twice.
(33) Permeabilize the cells with Triton X-100 (0.3%) for 20 min.
(34) Prepare the reaction buffer additive.
(35) Prepare the reaction cocktail according to the manufacture protocol [48] by adding the ingredients in the listed order. This cocktail solution should be used within 15 min of preparation.
(36) Wash cells with 1 mL 3% BSA twice.
(37) Add 500 μL reaction cocktail to each cell dish, incubate for 30 min in dark.
(38) Wash cells with 1 mL 3% BSA once.
(39) Follow single cell staining steps (8)−(25). If no AB staining needed (only EdU staining), proceed with steps (21)−(25).
